# Periostin Links Skin Inflammation to Melanoma Progression in Humans and Mice

**DOI:** 10.3390/ijms20010169

**Published:** 2019-01-04

**Authors:** Fumitaka Ohno, Takeshi Nakahara, Makiko Kido-Nakahara, Takamichi Ito, Satoshi Nunomura, Kenji Izuhara, Masutaka Furue

**Affiliations:** 1 Department of Dermatology, Graduate School of Medical Sciences, Kyushu University, Fukuoka 812-8582, Japan; fumi16@med.kyushu-u.ac.jp (F.O.); macky@dermatol.med.kyushu-u.ac.jp (M.K.-N.); takamiti@dermatol.med.kyushu-u.ac.jp (T.I.); furue@dermatol.med.kyushu-u.ac.jp (M.F.); 2 Division of Skin Surface Sensing, Graduate School of Medical Sciences, Kyushu University, Fukuoka 812-8582, Japan; 3 Division of Medical Biochemistry, Department of Biomolecular Sciences, Saga Medical School, Saga 849-8501, Japan; nunomura@cc.saga-u.ac.jp (S.N.); kizuhara@cc.saga-u.ac.jp (K.I.); 4 Research and Clinical Center for Yusho and Dioxin, Kyushu University Hospital, Fukuoka 812-8582, Japan

**Keywords:** periostin, M2 macrophages, prognosis, melanoma, inflammation

## Abstract

It is widely accepted that chronic inflammation initiates and promotes carcinogenesis and tumor progression in various cell types. However, this paradigm has not been comprehensively investigated in melanoma. To investigate the effects of chronic inflammation on the progression of melanoma, we established a murine inflammatory skin model and investigated the relationship between skin inflammation and melanoma progression. In a murine model, B16F10 melanoma cells in inflamed skin grew significantly more rapidly than cells in control skin. The stromal expression of periostin was upregulated in inflamed skin, and significantly more CD163^+^ M2 macrophages were recruited to the melanomas in inflamed skin. We then immunohistologically examined the expression of stromal periostin and the infiltration of CD163^+^ M2 macrophages in human acral lentiginous melanomas (*n* = 94) and analyzed the statistical associations with clinicopathological variables. In human melanomas, high periostin expression and a large number of infiltrated M2 macrophages were significantly correlated with poor prognosis. Furthermore, we confirmed that periostin promotes the proliferation of murine and human melanoma cells in vitro. Our findings indicate that periostin and M2 macrophages play a critical role in melanoma progression and prognosis in both humans and mice, indicating that periostin is a potential target for treating progressive melanoma.

## 1. Introduction

Inflammation is recognized as a hallmark of cancer development and progression [1,2,3]. Melanomas comprise heterogeneous groups, for example, cutaneous, uveal, acral, and mucosal melanomas, depending on the clinical distribution [4,5]. Individuals with white skin (Fitzpatrick skin types I and II) have a higher likelihood of developing cutaneous melanomas than those with darker skin, which is likely related to exposure to ultraviolet rays [6]. In Asian populations, melanomas commonly occur in the palmoplantar region, given that this area is burdened with frequent chemical contact, friction, and body pressure [4,5,7]. Although the role of inflammation in cancer progression has been extensively studied [1,2,3], the association between inflammation and melanoma progression is not well-understood.

In the inflammatory tumor microenvironment of melanoma, many factors such as immune cells, extracellular matrix protein, and cytokines can influence melanoma progression [1,2,3]. For instance, increased expression levels of cyclooxygenase-2 (COX-2), which has emerged as the key enzyme regulating inflammation, were correlated with the development and progression of human melanoma. Moreover, it was reported that melanoma cells that expressed a higher level of COX-2 also co-expressed a higher level of programmed death ligand 1 (PD-L1). These reports supported the critical involvement of inflammation in melanoma progression. [8,9] Thus, we first investigated the effects of skin inflammation on melanoma progression using a murine chronic inflammation model. We also determined important factors that link skin inflammation to melanoma progression in the model. In the murine model, accelerated B16F10 melanoma growth was significantly associated with the intensity of periostin expression and the number of infiltrated M2 macrophages. We then immunohistologically examined the expression of periostin and infiltrated M2 macrophages in human melanomas, and demonstrated that the intensity of periostin expression was indeed inversely correlated with prognosis in patients with melanoma. The intensity of stromal periostin expression was also significantly associated with the number of infiltrated M2 macrophages, and the number of infiltrated M2 macrophages was associated with the poor prognosis of melanoma patients. These findings indicate that periostin plays a critical role in human and murine melanoma progression and the prognosis of patients with melanoma.

## 2. Results

### 2.1. Chronic Inflammation Significantly Accelerates Murine Melanoma Progression

**Figure 1:**
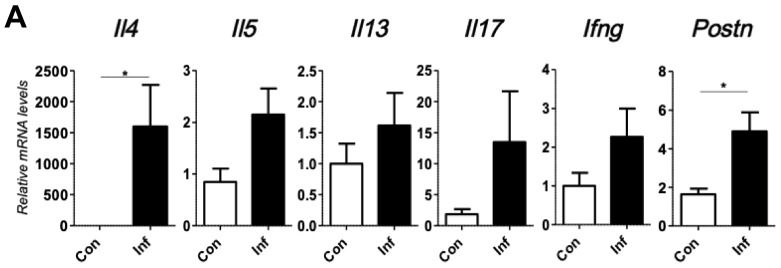

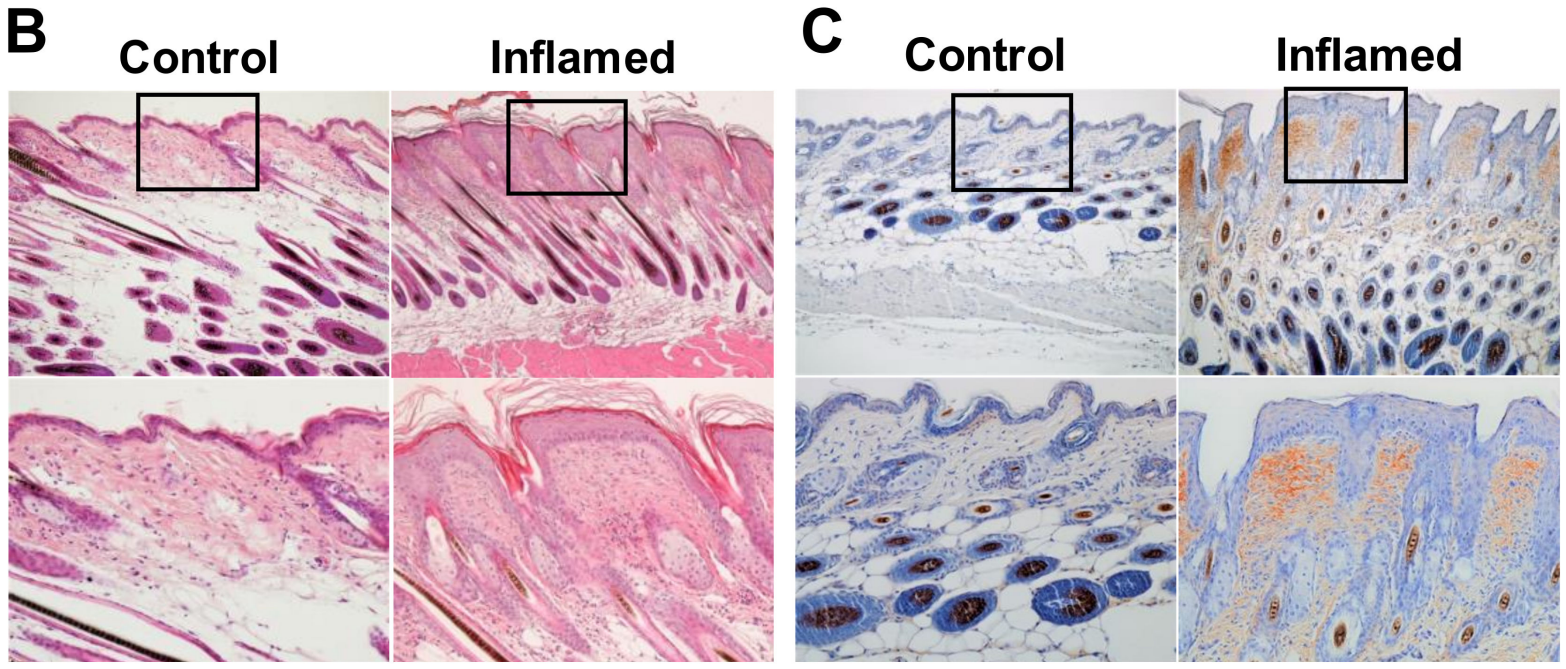
Transcriptional and immunohistochemical analyses of a murine chronic inflammation model. After inflammation or control treatment, all mice were sacrificed. Then the skin tissues were harvested for qRT-PCR and immunohistochemical analysis. (**A**) mRNA expression of inflammatory cytokines (*Il4*, *Il5*, *Il13*, *Il17*, *Ifng*, *periostin*) in control and inflamed mice. (**B**) Hematoxylin-eosin staining of skin in control and inflamed mice. Upper; ×40 magnification. Lower; ×100 magnification. (**C**) Immunohistochemical staining of periostin in control and inflamed mice. Upper; ×40 magnification. Lower; ×100 magnification. *t*-test; * *p* < 0.05. Each group consisted of eight mice. Representative data of three independent experiments are shown.

**Figure 2:**
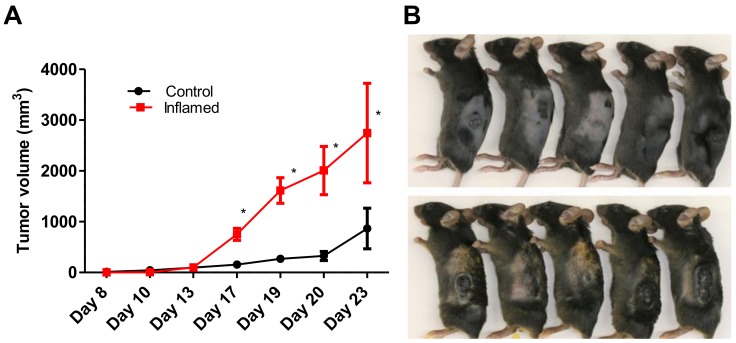
Chronic inflammation significantly accelerates murine melanoma progression. Control mice and inflamed mice were inoculated with B16F10 cells (1 × 10^5^ cells/100 µL of PBS) into the back skin. (**A**) Tumor volume was measured approximately every two days after B16F10 inoculation and was calculated as described in Materials and Methods. *t*-test; * *p* < 0.05. (**B**) Representative images of melanoma are shown. Upper panel: Control mice. Lower panel: Inflamed mice. Each group consisted of eight mice. Representative data of three independent experiments are shown.

To investigate the relationship between skin inflammation and melanoma progression, we established a murine inflammatory skin model. For this, we topically applied 1% trinitrochlorobenzene to the shaved back skin of C57/BL6 mice every other day for two weeks. This procedure successfully induced chronic skin inflammation with upregulated transcription of *Il4*, *Il5*, *Il13*, *Il17*, *Ifng*, and *periostin* (Figure 1A). Histopathologically, the inflamed skin showed significantly thicker epidermis, thicker dermal fibrosis, and greater inflammatory cell infiltration composed of mononuclear cells, eosinophils, and neutrophils compared to that observed in untreated normal skin (Figure 1B). In addition, the stromal expression of periostin was upregulated immunohistochemically in the inflamed skin (Figure 1C). We then inoculated B16F10 melanoma cells intradermally in the back skin of control or inflamed mice. Tumorigenesis of inoculated B16F10 melanoma cells was recognized around day 8 after inoculation. Notably, the melanoma tumor size became significantly larger on days 17 to 23 in the inflamed mice than that in the control mice (Figure 2). These results suggested that inflammation is potentially involved in driving melanoma progression.

### 2.2. Stromal Periostin Expression and the Number of CD163^+^ M2 Macrophages Are Significantly Elevated in Melanomas in Inflamed Mice

**Figure 3:**
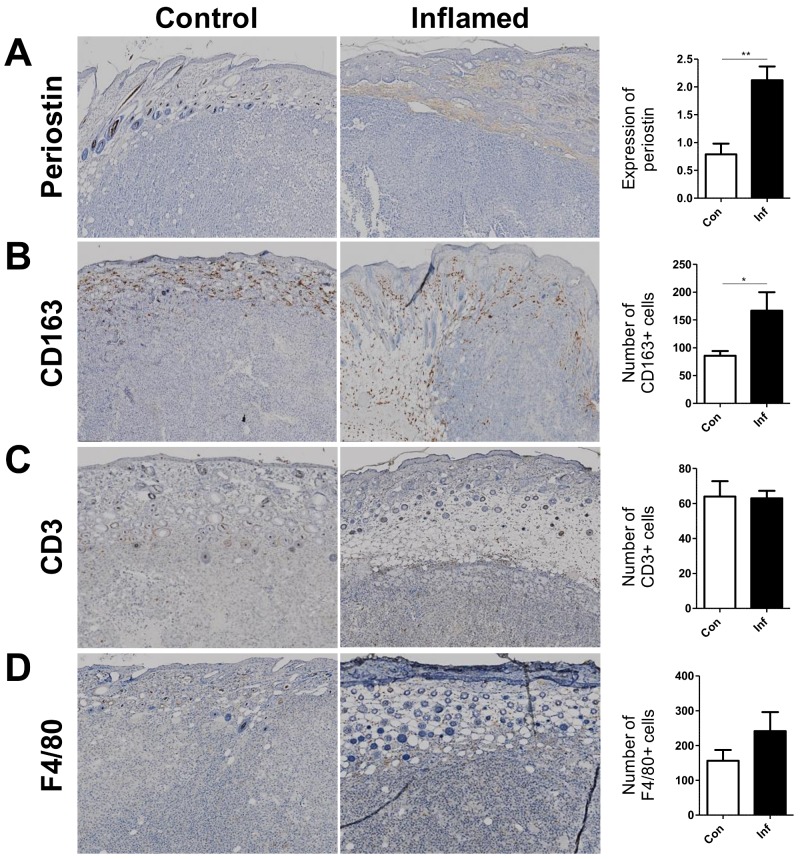
Stromal expression of periostin and the number of CD163^+^ M2 macrophages are significantly elevated in melanomas in inflamed mice, but not the number of CD3^+^ cells and F4/80^+^ cells. Left panels show representative immunohistochemical staining of (**A**) periostin, (**B**) CD163, (**C**) CD3, and (**D**) F4/80 in melanomas in control and inflamed mice. ×100 magnification. Right panels show scoring of the expression of periostin and the number of infiltrated CD163^+^ cells, CD3^+^ cells, F4/80^+^ cells. *t*-test, * *p* < 0.05, ** *p* < 0.01.

We next compared the expression of periostin and infiltrated cells in melanomas developed in control and inflamed skin. During melanoma tumorigenesis, small but detectable amounts of stromal periostin were detected by immunohistochemistry, even in the control mice. Notably, the stromal expression of periostin was significantly enhanced in the melanomas developed in inflamed mice compared with those in the control mice (Figure 3A). A significantly higher number of CD163^+^ M2 macrophages also infiltrated into the melanomas in the inflamed mice than that in the control mice (Figure 3B). This was a rather specific phenomenon because the numbers of infiltrated CD3^+^ T cells and F4/80^+^ macrophages were comparable between melanomas developed in inflamed mice and control mice (Figure 3C,D). These findings suggested that periostin and M2 macrophages might be factors that link skin inflammation and melanoma progression in our murine model.

### 2.3. High Periostin Expression and a Large Number of Infiltrated CD163^+^ M2 Macrophages Are Significantly Correlated with Poor Prognosis in Patients with Melanoma

**Figure 4:**
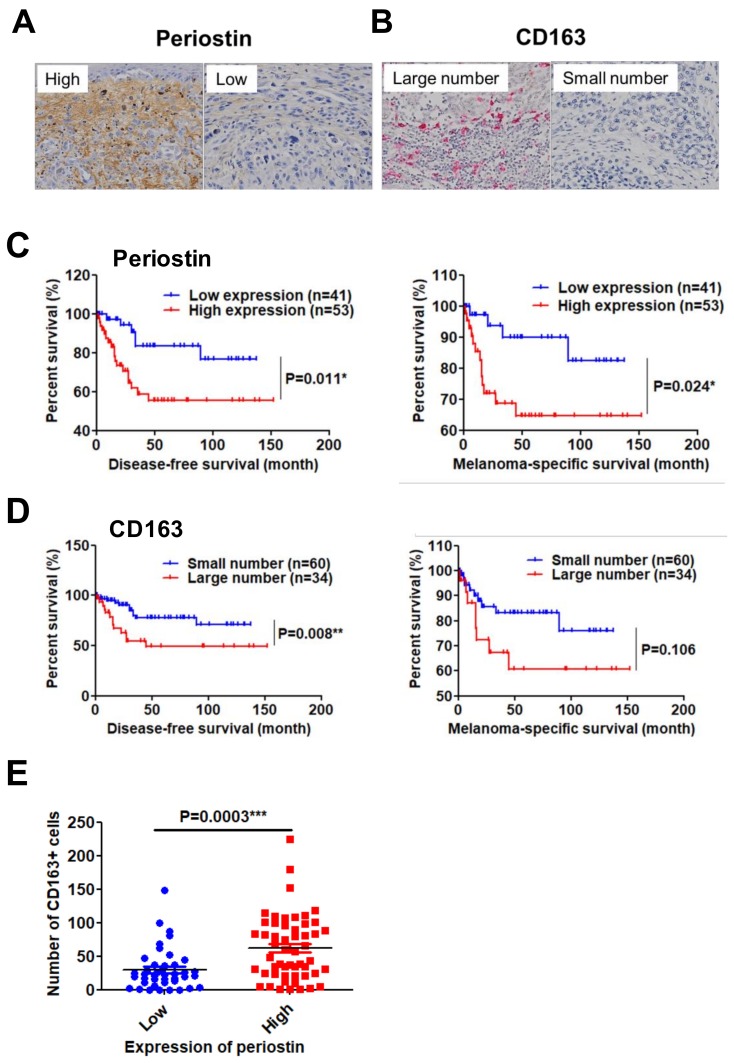
High periostin expression and a large number of infiltrated M2 macrophages are significantly correlated with poor prognosis in patients with melanoma. (**A**,**B**) Representative immunohistochemical staining of periostin (**A**) (brown) and CD163 (**B**) (red) in human melanomas. ×200 magnification. (**C**,**D**) Kaplan-Meier curve of 94 patients with melanomas regarding periostin (**C**) and CD163 (**D**) for DFS and MSS. *p*-values were obtained from the log-rank test (n, the number of samples in each group). * *p* < 0.05, ** *p* < 0.01. (**E**) Scatter diagram shows the relationship between the expression of periostin and the number of CD163^+^ M2 macrophages. Cut-off values for high/low expression of periostin and the number of CD163^+^ cells are described in the Results. *t*-test, *** *p* < 0.001.

The results of the animal study mentioned above suggested that periostin expression and CD163^+^ M2 macrophage infiltration might also be the prognostic factors for human melanoma. Therefore, we immunohistologically examined the expression of stromal periostin and the infiltration of CD163^+^ M2 macrophages in 94 melanoma samples, and statistically analyzed the associations of these variables with the patients’ histological features, clinical stage, and prognosis (Table S1). In the present study, the stromal expression of periostin was divided into high- and low-intensity groups (Figure 4A). The number of recruited CD163^+^ M2 macrophages was also divided into large-number (≥50 cells/high-power view) and small-number groups (≤49 cells/high-power view) (Figure 4B).

Among factors such as age, gender, Breslow thickness, and TNM stage, Breslow thickness and TNM stage were significantly correlated with the expression of periostin or the number of infiltrated M2 macrophages (Table 1). Kaplan-Meier analysis revealed that patients with high expression of periostin had significantly shorter disease-free survival (DFS) and melanoma-specific survival (MSS) (DFS: hazard ratio (HR) 2.832, 95% confidence interval (CI) 1.267–6.329, *p* = 0.011; MSS: HR 2.980, 95% CI 1.148–7.741, *p* = 0.013) (Figure 4C). Similarly, patients with a large number of CD163^+^ M2 macrophages had significantly shorter DFS (DFS: HR 3.264, 95% CI 1.356–7.857, *p* = 0.008; MSS: HR 2.370, 95% CI 0.832–6.747, *p* = 0.106) (Figure 4D).

In addition, patients with high periostin expression had a significantly higher number of CD163^+^ M2 macrophages (Figure 4E). These findings indicated that periostin and M2 macrophages are critical prognostic factors in patients with melanoma and may cooperatively promote melanoma progression.

### 2.4. Periostin Promotes the Proliferation of Murine and Human Melanoma Cells In Vitro

**Figure 5:**
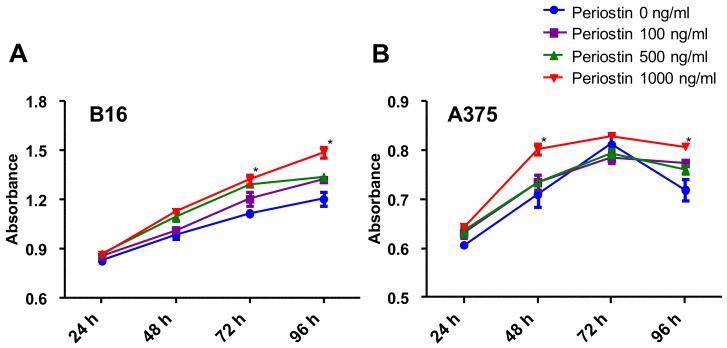
Proliferation assay of (**A**) B16F10 cells and (**B**) A375 cells. Cell proliferation was assessed by WST-1 assay every 24 h for 4 days. Cultures stimulated with periostin showed a significant increase in B16F10 cell number on days 3 and 4, and in A375 cell number on days 2 and 4. Three independent experiments were performed, and representative results are shown. *t*-test, * *p* < 0.05.

To verify the effect of periostin on the proliferation of melanoma cells, we next examined whether periostin could promote the cell growth of B16F10 murine melanoma cells and A375 human melanoma cells in vitro. Various doses of periostin (100 to 1000 ng/mL) significantly promoted the proliferation of B16F10 melanoma cells on day 3 and day 4 in a dose-dependent manner (Figure 5A). Periostin also slightly but significantly promoted the proliferation of A375 human melanoma cells on day 2 and day 4 (Figure 5B). These data support that periostin plays a critical role in melanoma progression in both mice and humans.

## 3. Discussion

Tumor growth and progression are influenced by the surrounding inflammatory milieu [1,3,10,11]. Besides the production of proinflammatory cytokines, the stromal response is important in inflammation-induced tumorigenesis or tumor-related inflammation [1,3,10,11]. Among the various stromal constituents, periostin has received increased attention in the context of allergic inflammation, immune tolerance, wound repair, and tumor progression [12,13,14,15]. Periostin, an extracellular matrix protein that belongs to the fasciclin family, functions as a matricellular protein in cell activation by binding to integrin receptors on the cell surface, thereby exerting its biological activities [12,13]. The accumulated knowledge about the relationship between periostin and cancer indicate that this protein modulates tumor cell behavior in many ways [14].

The pathological role of periostin in tumor progression remains controversial. Although periostin overexpression promotes the growth or motility of many types of tumor cells [14,15,16,17,18,19], its tumor-suppressing effects have also been observed [20,21]. The expression levels of periostin in melanoma are relatively high compared with those in other malignancies, which may be related to the fact that the skin is a site where periostin is highly expressed [19]. Even though melanoma cells are able to produce periostin, tumor-associated stromal fibroblasts are the major source of its production [15,19,22]. Moreover, the histological invasiveness and lymph node metastasis of melanoma are significantly associated with stromal periostin expression [15,19,22]. Intriguingly, periostin facilitates the recruitment of CD163^+^ M2 macrophages, which potently induces immune tolerance and promotes tumor progression [23]. However, no studies have comprehensively investigated the relationships between periostin and CD163^+^ M2 macrophages regarding the progression and prognosis in patients with melanoma.

In our murine study, skin inflammation significantly promoted melanoma growth. The B16F10 melanoma cells injected into inflamed skin grew significantly faster than those in control mice. The stromal expression of periostin was significantly enhanced in the melanomas developed in the inflamed mice compared with those in the control mice. The melanomas in the inflamed skin harbored more CD163^+^ M2 macrophages. These results of murine experiments highlighted the importance of inflammation-induced periostin and CD163^+^ M2 macrophage infiltration in melanoma progression. This led us to investigate if periostin and CD163^+^ M2 macrophage infiltration were prognostic factors in human melanoma. In our study of human melanoma, periostin was highly expressed in the stroma of thick melanomas. In addition, a larger number of CD163^+^ M2 macrophages infiltrated into thick melanomas than into the thin ones. Notably, these markers predicted worse prognosis of patients with melanoma.

The present clinical and immunohistological findings coincided well with the previous reports describing that periostin was overexpressed in the stroma in invasive and metastatic melanoma lesions in humans [15,19,22]. In a study of a previously established mouse model, it was shown that wounding as well as subcutaneously injected osteoblasts that secrete large amounts of periostin promoted the metastasis of remotely transplanted melanoma cells [15], which also mirrored our murine result showing that periostin-mediated skin inflammation augmented melanoma expansion. A recent study using B16F10 melanoma cells deficient in CCN2 (connective tissue growth factor) also highlighted the importance of periostin for melanoma metastasis [24].

The recruitment of M2 macrophages has also been documented to indicate poor patient outcome [25]. Our results also confirmed that a high number of CD163^+^ M2 macrophages was associated with worse patient prognosis.

In conclusion, stromal periostin and the number of CD163^+^ M2 macrophages are mutually correlated risk factors for a poor outcome in patients with melanoma. Targeting the interaction of periostin with other proteins may be a promising strategy for developing new therapies for melanoma.

## 4. Materials and Methods

### 4.1. Mice

C57/BL6 female mice, aged six to seven weeks, were obtained from Charles River Laboratories. The mice were maintained in a flexible vinyl film isolator. All experiments were approved by the Animal Care and Experiments Committee of Kyushu University.

### 4.2. In Vivo Experiments

For the in vivo experiments, 1% trinitrochlorobenzene (C0307; Tokyo Chemical Industry, Tokyo, Japan) was topically applied to the shaved back skin of each mouse every other day for two weeks (18). The vehicle was applied to control mice. We then inoculated B16F10 cells (1 × 10^5^ cells/100 µL of PBS) into the back skin intradermally. Tumor growth was monitored for three weeks by measuring the major and minor axes of the tumor using calipers. Tumor volume was calculated using the following formula: (major diameter × minor diameter^2^)/2.

### 4.3. Quantitative RT-PCR Analysis

Total RNA was isolated from skin and solid tumors using TRIzol Reagent (15596018; Life Technologies Corporation, Carlsbad, CA, USA) and extracted with an RNeasy Mini kit (74104; Qiagen, Valencia, CA, USA). Reverse transcription was performed with a PrimeScript RT-reagent kit (RR037A; Takara Bio, Shiga, Japan). qRT-PCR was performed on a CFX Connect Real-time system (185-5201J1; Bio-Rad, Hercules, CA, USA) with SYBR Premix Ex Taq (RR820A; Takara Bio). The PCR programming was initiated at 95 °C for 30 s, followed by 40 cycles of qRT-PCR at 95 °C for 5 s and 60 °C for 20 s. Levels of mRNA expression were estimated from the fluorescence intensity using the level of β-actin for normalization. The primers, from Hokkaido System Science, were as follows: *Il4* (forward 5′-GAATGTACCAGGAGCCATATC-3′, reverse 5′-CTCAGTACTACGAGTAATCCA-3′), *Il5* (forward 5′-TCACCGAGCTCTGTTGACAA-3′, reverse 5′-CCACACTTCTCTTTTTGGCG-3′), *Il13* (forward 5′-ACCCAGAGGATATTGCATGG-3′, reverse 5′-TGGGCTACTTCGATTTTGGT-3′), *Il17a* (forward 5′-TTTAACTCCCTTGGCGCAAAA-3′, reverse 5′-CTTTCCCTCCGCATTGACAC-3′), *Ifng* (forward 5′-CGGCACAGTCATTGAAAGCCTA-3′, reverse 5′-GTTGCTGATGGCCTGATTGTC-3′), and *Postn* (forward 5′-AAGCTGCGGCAAGACAAG-3′, reverse 5′-TCAAATCTGCAGCTTCAAGG-3′).

### 4.4. Immunohistochemistry and Scoring

All tissue samples were fixed with 10% formalin, embedded in paraffin, and sectioned at a thickness of 3 µm. Sections were deparaffinized with xylene for 10 min and rehydrated through a graded ethanol series. Antigen retrieval was performed using heat processor solution pH 6.0 (Nichirei Biosciences Inc., Tokyo, Japan) or 9.0 (Nichirei Bioscience Inc.) at 100 °C for 40 min, and endogenous peroxidase was blocked by incubating the sections with 3% H_2_O_2_ (Nichirei Biosciences Inc.). For immunohistochemical staining, primary antibodies were used at the following dilutions: human and murine anti-periostin (0.3 µg/mL, clone no. SS19C), human and murine anti-CD163 (1:500, ab182422; Abcam, Cambridge, UK), murine anti-CD3 (1:100, ab5690; Abcam), murine anti-F4/80 (1:100, ab100790; Abcam). All samples were incubated with secondary antibody, N-Histofine Simple Stain MAX-PO MULTI (Nichirei Biosciences Inc.). Immunodetection was conducted with 3,3′-diaminobenzidine (Nichirei Biosciences Inc.) or Fast Red kit (Nichirei Biosciences Inc.) as a chromogen, followed by light counterstaining with Giemsa or hematoxylin. Immunostaining of periostin in the murine model was stratified into three categories: 0, no expression; 1+, moderate; 2+, strong (Figure S1). To evaluate the expression of periostin, CD163, CD3, and F4/80, three high-power field images (HPFs, ×100) were randomly selected in each specimen, then periostin expression was scored and the number of CD163^+^, CD3^+^, and F4/80^+^ cells was counted. The sections were scored by two independent observers in a blinded fashion.

### 4.5. Patients and Tissue Samples

This study is a retrospective review of our patients conducted in accordance with the principles embodied in the Declaration of Helsinki, and was approved by the Institutional Ethics Committee of Kyushu University (No. 26-1). We analyzed 94 patients with acral lentiginous melanoma at the Department of Dermatology, Kyushu University Hospital, Fukuoka, Japan, between 2001 and 2014. All patients were Japanese with a mean age of 65.6 years. Their demographic data, including age, gender, Breslow thickness, TNM stage, tumor site, the presence of ulceration, melanoma-specific survival (MSS), and disease-free survival (DFS) are described in Supplementary Table S1. DFS and MSS were calculated from the date of the first histopathological examination to the date of recurrence or the date of death as a result of melanoma. Data on patients without death or recurrence were censored on the date of the last follow-up before March 31, 2014, and data on patients who died of other causes were censored at the time of death. 

### 4.6. Cell Culture

The B16F10 murine melanoma cell line and A375 human melanoma cell line were obtained from the American Type Culture Collection. B16F10 cells and A375 cells were cultured in Dulbecco’s Modified Eagle’s Medium (D5796; Sigma Chemicals, St. Lousi, MO, USA) supplemented with 1% Modified Eagle’s Medium Non-Essential Amino Acids (11140050; Life Technologies, Carlsbad, CA, USA), 1% HEPES (15630080; Life Technologies), 1% sodium pyruvate (11360070; Life Technologies), 5% fetal bovine serum (CCP-FBS-BR-500; Cosmo Bio, Tokyo, Japan), 2 mM L-glutamine, 100 units/mL penicillin, and 10 µg/mL streptomycin (10378016; Life Technologies).

### 4.7. Proliferation Assay

Cell proliferation of B16F10 murine melanoma cells and A375 human melanoma cells was analyzed using Cell Proliferation Reagent WST-1 and the absorbance at 450 nm. The cells were seeded in triplicate at a density of 5000 cells in 200 µL of starvation culture medium lacking FBS in 96-well plates for 1, 2, 3, or 4 days. Each medium contained a different concentration of periostin (100 to 1000 ng/mL). After treatment with different concentrations of periostin, 20 µL WST-1 reagent was added to each well, and the plates were incubated 37 °C for 3 h at each time point.

### 4.8. Statistical Analysis

All statistical analyses were performed using the SPSS 11 statistical software package (IBM Corp., Armonk, NY, USA) and the GraphPad Prism 5 statistical software package (GraphPad Software Inc., La Jolla, CA, USA). The associations between periostin expression, the number of CD163^+^ M2 macrophages, and clinicopathological parameters were assessed using the χ^2^ test or Fisher’s exact test. The Kaplan-Meier method was used to estimate DFS and MSS, and the log-rank test was used to evaluate the association between survival and the expression of periostin and/or the number of CD163^+^ M2 macrophages. Comparisons of the associations between the expression of periostin and the number of CD163^+^ M2 macrophages in human samples, as well as the results of murine tumorigenic assay between the two groups (control mice vs. inflamed mice), were performed by unpaired Student’s *t*-test. A *p*-value <0.05 was considered to indicate a statistically significant difference.

## Figures and Tables

**Table 1:** Associations between the expression of periostin, the number of CD163^+^ cells, and clinicopathological factors.

Parameters	Expression of Periostin			Number of CD163^+^ Cells		
	Low	High	*p*-Value	Small	Large	*p*-Value
Age (y)						
<60	15	10	0.053 ^¶^	18	5	0.13 ^∫^
≥60	26	43		42	29	
Gender						
Male	16	24	0.54 ^¶^	23	17	0.27 ^¶^
Female	25	29		37	17	
Breslow Thickness ^§^						
Tis	20 **	4 ^††^	<0.0001 ^‡^^¶^	23 **	1 ^††^	0.0001 ^‡^^¶^
T1	8	9		14	3	
T2	5	2		4	3	
T3	6	15		8 ^††^	13 **	
T4	2 ^††^	22 **		10 ^†^	14 *	
TNM Stage						
0	20 **	4 ^††^	<0.0001 ^‡^^¶^	23 **	1 ^††^	<0.0001 ^‡^^¶^
I	11	10		17	4	
II	4 ^††^	22 **		13	23	
III	6	12		5^††^	13 **	
IV	0 ^†^	5 *		2	3	
Total No.	41	53		60	34	

* *p* < 0.05 significantly high, ** *p* < 0.01 significantly high, † *p* < 0.05 significantly low, †† *p* < 0.01 significantly low, ¶ χ^2^ test, ∫ Fisher’s exact test, § available data only, ^‡^ significant values.

## Supplementary Materials

Supplementary materials can be found at http://www.mdpi.com/1422-0067/20/1/169/s1.

## Abbreviations

TGF	transforming growth factor
IL	Interleukin
PDGF	platelet-derived growth factor
CCN	connective tissue growth factor
DFS	disease-free survival
