# Supplementary material for: Periostin Links Skin Inflammation to Melanoma Progression in Humans and Mice
Source: Int J Mol Sci. 2019 Jan 4;20(1):169. doi: 10.3390/ijms20010169 (PMC6337622; doi:10.3390/ijms20010169)
Supplement: Supplementary file 1 [file ijms-20-00169-s001.pdf]

Supplementary Materials

Supplementary Table S1. Patient demographic and clinical data.

Parameters	
Age, y	
Range (mean ± SD)	32-88 (65.6 ± 14.2)
Gender, No (%)	
Male	40 (42.6)
Female	54 (57.4)
Breslow thickness, No (%)	
Tis	24 (25.8)
T1	17 (18.3)
T2	7 (7.5)
T3	21 (22.6)
T4	24 (25.8)
TNM stage, No (%)	
0	24 (25.8)
I	21 (22.3)
II	26 (27.7)
III	18 (19.1)
IV	5 (5.3)
MSS, mo	
Range (mean ± SD)	0-152 (48.2 ± 44.6)
DFS, mo	
Range (mean ± SD)	0-152 (45.8 ± 43.7)
Total patients	94

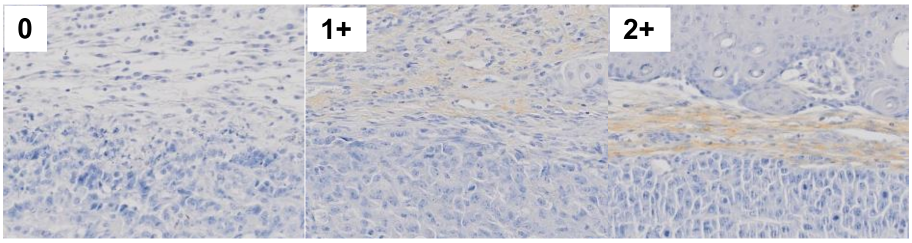

Supplementary Figure S1. Immunohistochemical scoring pattern of periostin staining in murine melanoma.
